# STAT inhibitors for cancer therapy

**DOI:** 10.1186/1756-8722-6-90

**Published:** 2013-12-05

**Authors:** Muhammad Furqan, Akintunde Akinleye, Nikhil Mukhi, Varun Mittal, Yamei Chen, Delong Liu

**Affiliations:** 1 Department of Medicine, New York Medical College and Westchester Medical Center, Valhalla, NY 10595, USA; 2 Department of Medicine, SUNY Downstate Medical Center Brooklyn, Brooklyn, NY 11203, USA; 3 Department of Hematology, Xiamen Zhongshan Hospital, Xiamen University, Xiamen, China; 4 Division of Hematology and Oncology, Department of Medicine, New York Medical College and Westchester Medical Center, Valhalla, NY 10595, USA

## Abstract

Signal Transducer and Activator of Transcription (STAT) proteins are a family of cytoplasmic transcription factors consisting of 7 members, STAT1 to STAT6, including STAT5a and STAT5b. STAT proteins are thought to be ideal targets for anti-cancer therapy since cancer cells are more dependent on the STAT activity than their normal counterparts. Inhibitors targeting STAT3 and STAT5 have been developed. These included peptidomimetics, small molecule inhibitors and oligonucleotides. This review summarized advances in preclinical and clinical development of these compounds.

## Introduction

Signal Transducer and Activator of Transcription (STAT) proteins are a family of cytoplasmic transcription factors consisting of 7 members, STAT1 to STAT6, STAT5a and STAT5b [1]. They are activated either by receptor associated tyrosine kinases like Janus kinases (JAKs) or by receptors with intrinsic tyrosine kinase activity e.g. PDGFR, EGFR, FLT3. They can also be activated by constitutively active non-receptor protein tyrosine kinases (PTKs), such as c-Src Bcr-Abl, and Brk (Breast tumor kinase) [2,3]. Specific phosphorylation of STAT proteins by these tyrosine kinases causes their homo- or hetero-dimerization. These dimers then migrate to the nucleus to control gene expression.

Each STAT protein is encoded by a separate gene. However they share structural similarities with six conserved domains. These conserved domains from N to C terminus include oligomerization domain, coiled coil, DNA binding domain, linker domain, SH2 domain, and transactivation domain. Alternate mRNA splicing or proteolytic processes can give rise to multiple isoforms lacking part of the c-terminal domain and referred to as STATβ isoforms as opposed to regular full length STATα. These truncated β isoforms put forth dominant negative effect and compete with regular α isoforms. β isoforms are isolated in case of STAT1, 3 and 5 [1].

Functionally STAT2, 4, & 6 regulate immune responses whereas STAT1, 3, & 5 have diverse physiological role. They regulate expression of genes controlling cell cycle (*Cyclin D1, D2, and c-Myc*), cell survival (*Bcl-xL, Bcl-2, Mcl-1*), angiogenesis (*HIF1α, VEGF*) etc. Because of their critical role they are precisely regulated with activation and deactivation cycle of no more than a few hours [1]. Dysregulation at any level, i.e. cytokines, growth factors, tyrosine kinases, negative feedback mechanisms involving STAT pathway, can lead to increase in their activity and contribute to tumorigenesis.

STAT3 and 5 are persistently activated in many human cancer cell lines [2,4,5]. They are not only involved in cancer development and progression but also contribute to their survival [6]. Likewise, inhibition of STAT signaling induces growth arrest in several cancer models, suggesting their role as point of convergence from multiple upstream oncogenic pathways [5,7,8]. In addition, it has been demonstrated that cancer cells are more dependent on STAT activity than their normal counterparts. Several studies have illustrated to this fact that blocking STAT3 or 5 signaling lead to apoptosis in tumor cells. Whereas healthy cells were not only able to survive at very lower level of STAT3 or 5 but also capable of growing by an alternative mechanism [5]. Therefore targeting these transcriptional factors is very appealing in development of new anti-cancer therapy as it will block assembly of upstream molecular aberrations with less toxicity.

Aberrations of STAT3 signaling in various cancer models were appreciated before STAT5, therefore investigators initially concentrated on targeting STAT3. However, STAT5 emerged as an equally important activated transcription factor in many cancers [7,9,10]. Nonetheless, effort made in this regard is very little. Only few candidate molecules have been found to date that are specifically active against STAT5. This review will focus on progress of agents that directly inhibit STAT3 and STAT5 without significantly affecting other tyrosine kinases or SH-2 containing proteins. In addition these inhibitors do not possess appreciable influence on other cellular survival signaling pathways like MAPK/ERK, PI3K/mTOR/Akt.

### Peptides and peptidomimetics

**Figure 1:**
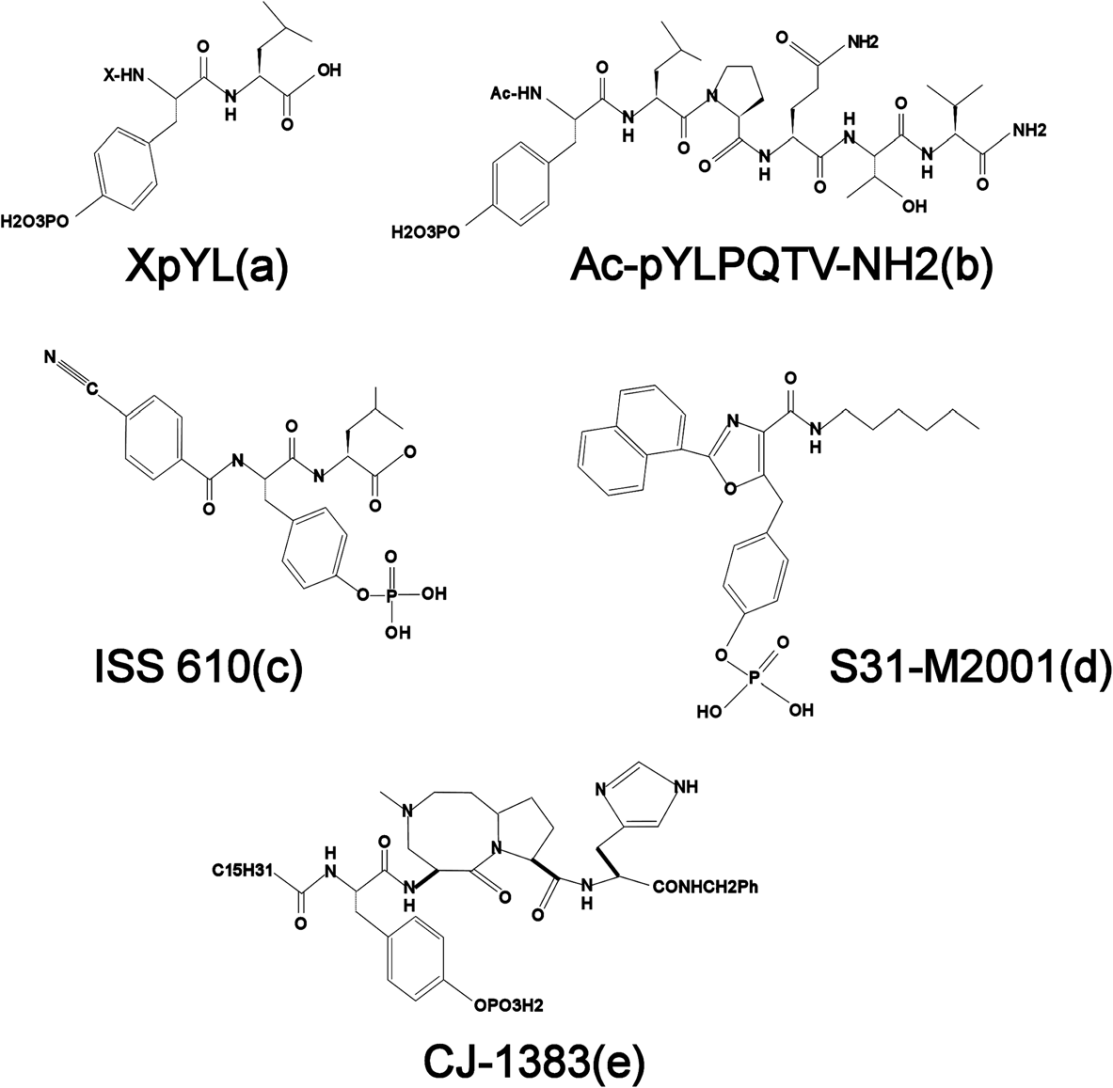
Chemical structures of peptides and peptidomimetics (a-e).

Turkson and his colleagues were the first to utilize peptides and its mimetic compounds to directly target STAT signaling. Their experiments *in vitro* and *in vivo* (Src-transformed NIH 3 T3/v-Src fibroblasts), showed that disrupting STAT3:STAT3 dimerization can effectively inhibit its transcriptional activity. They used STAT3 derived phospho-peptide, Pro-pTyr-Leu-Lys-Thr-Lys (PpYLKTK), which binds to native C-terminal STAT3-SH2 domain, to compete with phosphorylated STAT3 monomer and prevent their dimerization (IC_50_ = 235 μM) [11]. More importantly, they observed that phosphorylation of tyrosine residue, presence of Leucine at Y + 1 and a substituent at Y-1 were essential for the activity of this phospho-peptide. This particular composition of three amino acids (XpYL, Figure 1a) was the minimum phosphopeptide sequence required for its inhibitory activity.

STAT3 via its SH2 domain binds to phospho-tyrosine residue of several proteins like gp130, leukemia inhibitory factor receptor (LIFR), epidermal growth factor receptor (EGFR), interleukin 10 receptor (IL-10R), and granulocyte colony stimulating factor receptor (G-CSFR). Ren et al. develop another potent phosphopeptide from STAT binding sequence of gp130, Ac-pYLPQTV-NH3 (IC_50_ = 150nM: Figure 1b), having activity against STAT3. They also pinpointed that Leucine at pY + 1 and Glutamine at pY + 3 were critical for its activity [12].

Peptidomimetics have better pharmacokinetic properties than peptides. As a result investigators employed the afore-mentioned peptide, XpYL as the basic structural scaffold to develop their peptidomimetic compounds. Out of these, ISS610 (IC_50_ = 42 μM: Figure 1c) [13]; and S31-M2001 (IC_50_ = 79uM: Figure 1d) had superior pharmacokinetic profiles [14].

Similarly several other peptidomimetic molecules have been developed from the basic scaffold of compound shown in Figure 1b [15-19]. Among these CJ-1383 (Figure 1e) showed promising results with IC_50_ = 3–11 μM in two breast cancer cell lines containing high levels of phosphorylated STAT3 [15].

Despite hard work of several investigators, these agents need substantial improvement with regards to their *in vivo* metabolic susceptibility and cellular permeability prior to clinical testing. For the same reason no promising STAT5 dimerization inhibitor could be developed from this class (Figure 1).

### Non-peptidic small molecule inhibitors

Advances in medicinal chemistry, application of technology like high-throughput screening and desirable pharmacokinetic properties of small molecules led to increase in adoption of these agents for drug development. Indeed they constitute the largest class of STAT inhibitors at present (Table 1).

**Table 1:** ***In vitro *** **and *in vivo *** **studies of non-nucleotide based STAT inhibitors**

**Agent (Ref)**	**Cancer type studied *in vitro *** **(cell lines)**	**Cancer type studied *in vivo *** **(cell lines)**
Stattic (20, 31, 40, 42)	Breast (MDA-MB-231, -435S,-468, SUM159, SK-BR-3), Hepatocellular (SNU-387, -398, -449, Hep3B HepG2, Huh-7), Pancreatic (Panc-1, HPAC, BXPC-3, SW1990), Colon (SW480, HCT-116), Glioblastoma (U87, U251), Multiple Myeloma (U266, ARH77)	
STA-21 (22, 23)	Rhabdomyosarcoma (RH30, RD2), Breast (MDA-MB-231, -435 s, -468)	
LLL-3 (24,25)	Glioblastoma (U87, U251, U373), Chronic Myeloid Leukemia (K562), Prostate (DU145)	Glioblastoma (U87)
LLL12 (26–29, 46–47)	Medulloblastoma (Daoy, UW426, UW288-1), Glioblastoma (U87, U87delta, U373), Multiple Myeloma (U266, ARH-77 & patient’s primary cell lines), Breast (MDA-MB-231, SK-BR3), Pancreatic (HPAC, Panc-1), Rhabdomyosarcoma lines (RD2, RH28, RH30), Osteosarcoma (U2OS, SAOS2, SJSA)	Multiple Myeloma (ARH-77), Glioblastoma (U87), Breast (MDA-MB-231), Osteosarcoma (OS33)
XZH-5 (29–31)	Rhabdomyosarcoma (RD2, RH30, RH28), Breast (MDA-MB-231, SUM 159), Pancreatic (Panc-1, SW 1990), Hepatocellular (SNU-387, SNU-398, HepG2, Huh-7)	
S31-201 (32, 40)	Breast (MDA-MB-231, -435, -468), Multiple Myeloma (U266, ARH77)	Breast (MDA-MB-231)
SF-1066 (33,34)	Breast (MDA-MB-231, -468); Pancreatic (Panc-1), Prostate (DU145), Acute Myeloid Leukemia (OCI-AML-2)	Breast (MDA-MB-231)
SF-1087 (33)	Breast (MDA-MB-468), AML (OCI-AML-2), Prostate (DU145)	
17o (35)	Breast (MDA-MB-468), Prostate (DU145), Multiple Myeloma (JJN3)	
Cryptotanshinone (37)	Prostate (DU145), Breast (MDA-MB-468)	
FLL32 (40, 42, 44, 45, 46, 47)	Rhabdomyosarcoma (RD2, RH28, RH30); Renal (ACHN, SK-RC-45, -54, Caki), Melanoma (A375, Hs294T), HN-SCC (UM-SCC-29, -74B), Breast (MDA-MB-231,-468, SUM159, SK-BR-3), Pancreatic (Panc-1, HPAC, BXPC-3, SW1990); Colon (SW480, HCT-116), Glioblastoma (U87, U251), Multiple Myeloma (U266, ARH77), Hepatocellular (SNU-449, SNU-398, SNU-387, Hep3B), Osteosarcoma (U2OS, SAOS2, SJSA)	Breast (MDA-MB-231), Osteosarcoma (OS33), Pancreatic^*^ (Panc-1 in chicken embryo chorioallontoic membrane)
FLL62 (45)	Renal cell carcinoma (ACHN, SK-RC-45, SK-RC-54, Caki), Melanoma (A375, Hs294T)	
C188-9 (49)	Acute Myeloid Leukemia (Kasumi-1, THP-1, GDM-1, NB-4)	
LY5 (50)	Osteosarcoma (U2OS), Rhabdosarcoma (RH30, RD2), Ewing Sarcoma (EW8).	Breast (MDA-MB-231)
BP-1108 & BP-1075 (52)	Chronic Myeloid Leukemia (K562) and Acute Myeloid Leukemia (MV4-11)	
IS3 295 (54)	Breast (MDA-MB-231,-435,-468), Prostate (DU145), Pancreatic (Panc-1), MM (U266)	
Galiellalactone (55)	Prostate cancer (DU145, PC-3)	Prostate (DU145)
JQ1 (58)	T cell –Acute Lymphoblastic Leukemia	

*Abbreviations*: *Ref*, references.

### Inhibitors targeting STAT3-SH2 binding domain

Similar to peptidomimetics, small molecule inhibitors interact with STAT-SH2 domain and hamper STAT: STAT dimerization, nuclear translocation and transcriptional activity. Several investigators independently screened diverse chemical libraries by different methods to identify specific small molecules that can inhibit STAT3.

**Figure 2:**
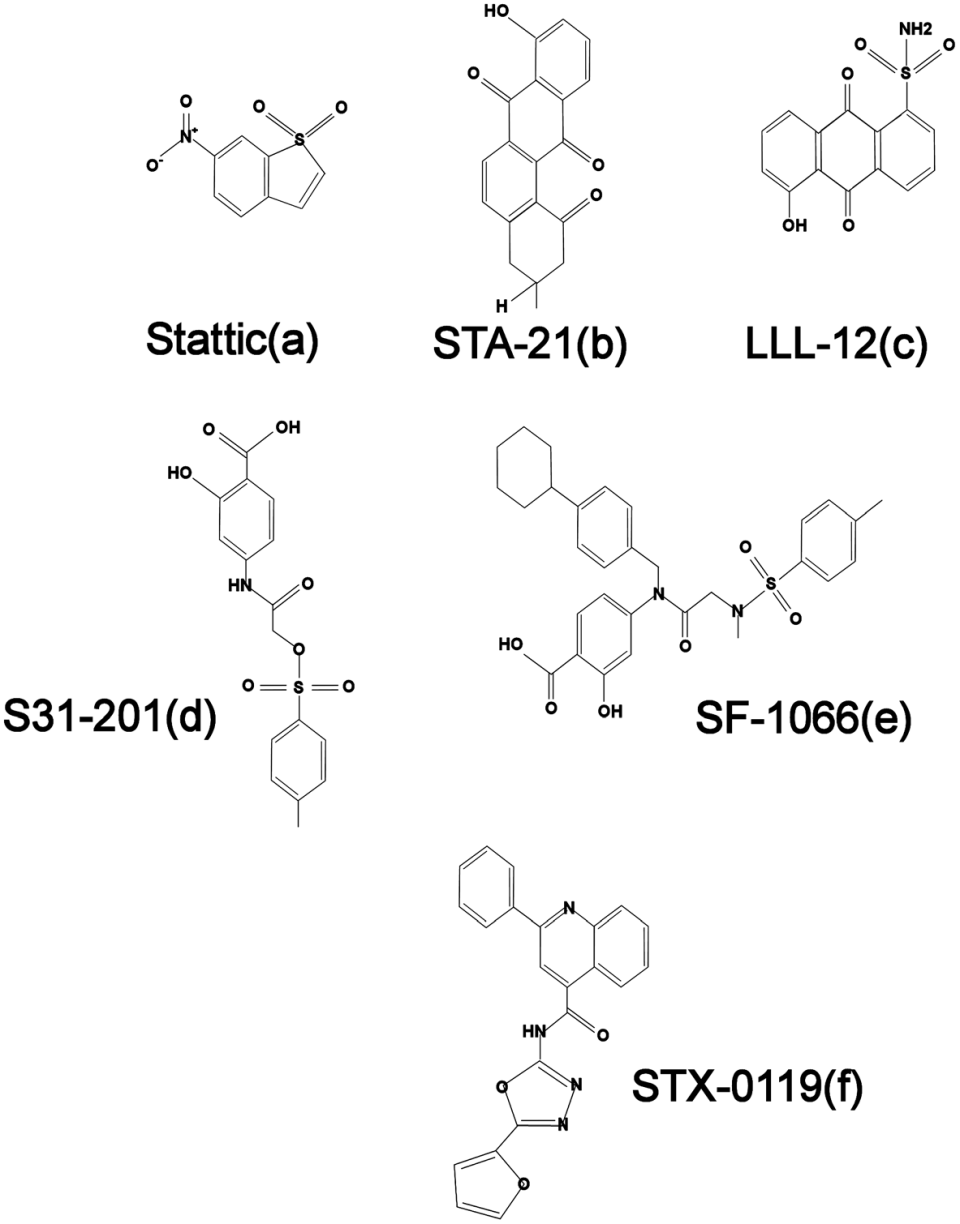
Chemical structures of small molecule inhibitors of STAT3 (a-f).

Stattic (STAT Three Inhibitory Compound: Figure 2a) was the first non-peptide small molecule discovered as inhibitor of STAT3 by high-throughput screening of diverse chemical libraries [20]. It selectively inhibits STAT3 dimerization relative to other members of STAT family. However, Sanseverino et al. recently questioned its selectivity against STAT3 [21]. It exhibited an IC_50_ of 5.1 μM in a fluorescence polarization assay and demonstrated increases in apoptotic rate of STAT3-dependent breast and hepatic cancer cells.

Lin and colleagues performed structure-based virtual screening of more than 425,000 compounds form four different chemical libraries to search a suitable STAT3 inhibitor. Out of top 200 compounds, they tested 100 chemicals with *in vitro* cell luciferase assay and found STA-21 (Figure 2b), a deoxytetrangomycin, as the most promising compound (IC_50_ of 12.2 μM & 18.7 μM in DU145 and PC3 cell lines respectively). It binds with SH2-domain of STAT3 and effectively inhibits STAT3 dimerization and demonstrated inhibition of growth and survival of breast and soft tissue sarcoma cell lines [22,23].

The same group developed a structural analogue of STA-21, LLL-3. This molecule has better cellular permeability than STA-21. Initially they treated human glioblastoma cell lines with LLL-3 *in vitro* and observed decreased viability of tumor cells (IC_50_ ranged between 10–15 μM in different Glioblastoma cell lines). The efficacy was also demonstrated by the observation that LLL-3 treated nude mice with intracranial glioblastoma lived longer than those treated with placebo [24]. Constitutive activation of STAT3 and STAT5 is observed in chronic myelogenous leukemic cells due to activity of Abl kinase, therefore Mencalha and colleagues treated K562 leukemic cells with LLL-3. They demonstrated that it decreased tumor cell survival and possesses synergistic effect with Imatinib [25].

For further optimization of LLL-3, Lin and colleagues replaced its acetyl group with sulfonamide and developed another STAT3 inhibitor, LLL12 (Figure 2c). It specifically prevents phosphorylation of Tyr 705 residue of STAT3 with IC_50_ ranging from 0.16 μM to 3.09 μM in various human cancer cell lines including HPAC, U87,U373, PANC1, and SK-BR-3 [26-28]. More recently, another non-peptide cell-permeable, small molecule, called as XZH-5, was studied. In the docking model, it binds with SH2-domain of STAT3 and prevents STAT3 phosphorylation at Tyr705, leading to inhibition of downstream STAT3 activities and apoptosis in multiple cancer cell lines including breast, pancreatic, hepatocellular carcinoma and rhabdomyosarcoma (IC_50_ ≈ 15-50 μM) [29-31].

Structure-based high-throughput virtual screening of the National Cancer Institute (NCI) chemical libraries identified another potent STAT3 inhibitor, S31-201. Its salicylic acid moiety docks with pTyr binding site of STAT3-SH2 domain. S31-201 (IC_50_ = 86 μM: Figure 2d) inhibited proliferation of hepatocellular and breast cancer cells in mice [32]. However GOLD docking studies suggested suboptimal interaction between STAT3 and S31-201. In an effort to improve this interaction, several molecules were rationally developed by Fletcher et al. Of these SF-1066 (Figure 2e) and SF-1087 are noteworthy with IC_50_ of 37 μM and 24 μM respectively in DU145 cell line [33,34]. They also reported 16 novel sulphoneamide analogues of SF-1066. Among those, 17o effectively inhibited STAT3:STAT3 interaction (IC_50_ = 19 μM) and was considered to be the most potent. Authors demonstrated that inhibition of STAT3 function in breast and myeloma cancer cells correlated with increased cell death (EC_50_ = 10 & 16 μM, respectively) [35].

Matsuno et al. recognized STX-0119 (IC_50_ = 74 μM: Figure 2f), a derivative of N-[2-(1, 3, 4-oxadiazolyl)]-4-quinolinecarboxamide by virtual screen using a customized version of DOCK4 program with the crystal structure of STAT3. Oral administrations of STX-0119 arrested the growth of human lymphoma cells in a SCC-3 subcutaneous xenograft model through inhibition of STAT3 activity [36].

Shin et al. searched within the natural compounds using a dual-luciferase assay to describe novel and specific inhibitor of STAT3. Cryptotanshinone, derived from roots of *Salvia miltiorrhiza* Bunge (Danshen, a Chinese herb), was identified as a potent STAT3 inhibitor. Cryptotanshinone inhibited STAT3 activity in a dose-dependent manner in HCT 116 colon cancer cells with an IC_50_ value of 4.6 μM. Activity of STAT3 was also inhibited in breast, prostate and cervical cancer cell lines. Study of binding mechanism revealed that cryptotanshinone directly interact with SH2 domain of STAT3 to inhibit Tyr705 phosphorylation and prevents STAT3 dimerization and nuclear translocation [37].

**Figure 3:**
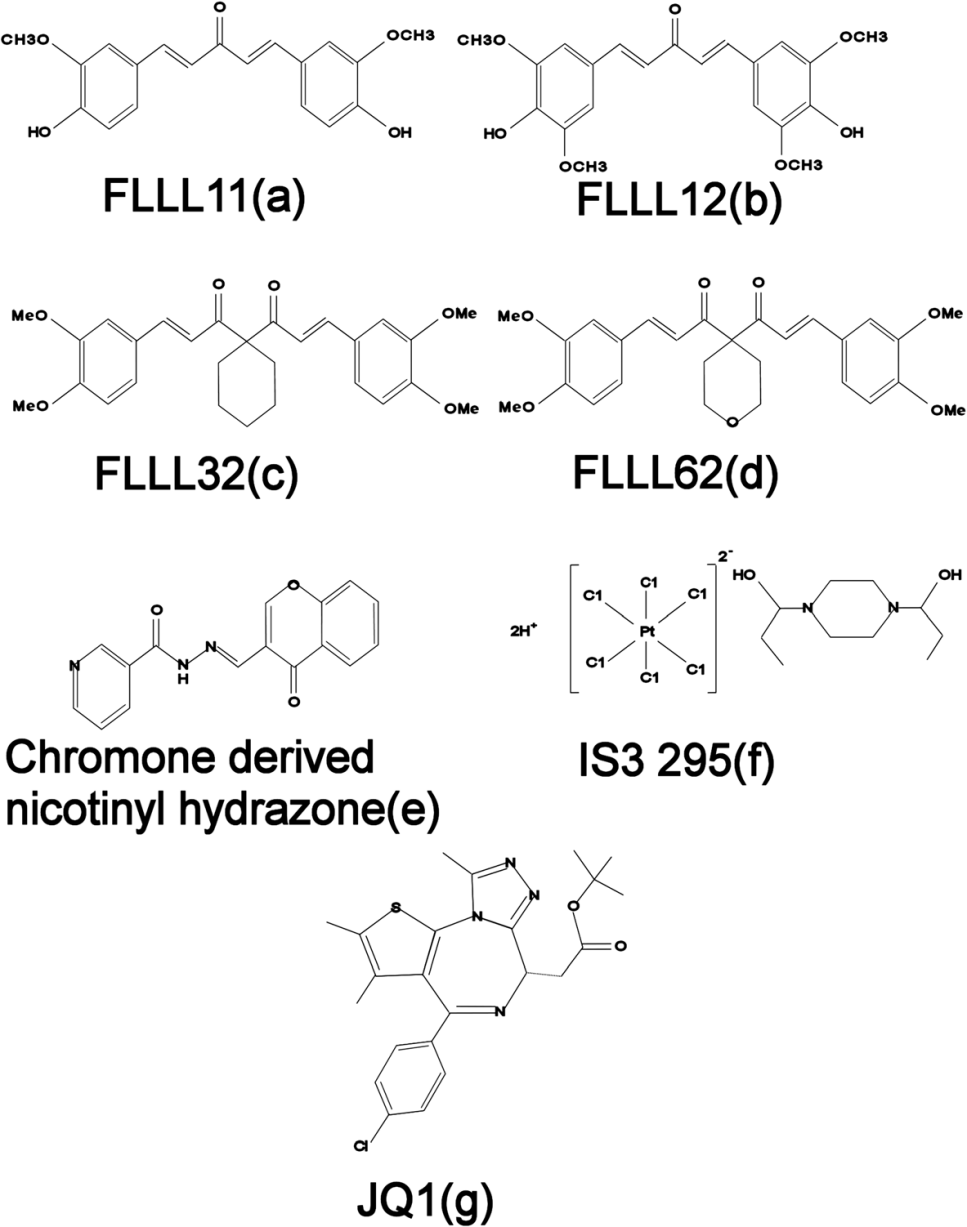
Chemical structures of small molecule inhibitors of STAT3 (a-d), STAT5 (e) and agents modulating their interactions with nuclear material (f, g).

Curcumin (diferuloylmethane; IC_50_ 20 μM in PC-3 prostate cancer cell lines), a component of the golden spice turmeric (*Curcuma longa*), can modulate multiple cell signaling pathways. Promising effects of this compound are seen in many conditions including cardiovascular diseases, arthritis, uveitis, inflammatory bowel disease, and in different types of cancers [38,39]. However, it is not readily absorbed from the gut after oral administration and has limited tissue distribution. This inspired many to develop compounds analogous to curcumin with better pharmacokinetics, including FLLL11 (IC_50_ 3.9 μM in PC-3 cell line: Figure 3a), FLLL12 ((IC_50_ 3.6 μM in PC-3 cell line: Figure 3b), FLLL32 (IC_50_ 4-8 μM in renal cancer cell lines: Figure 3c) and FLLL62 (IC_50_ 4-6 μM in renal cancer cell lines: Figure 3d). These analogues selectively bind to STAT3-SH2 domain to inhibit phosphorylation of Tyr705 and prevent its dimerization and downstream functioning. They are more potent than curcumin and shown to inhibit growth of many human cancer cell lines including pancreatic, breast, renal cell, hepatocellular, squamous cell cancer of head & neck region, colorectal, melanoma, myeloma, glioblastoma, osteosarcoma, and rhabdomyosarcoma [40-47].

One chemical library with 920,000 small drug-like compounds virtually screened by docking each into the peptide-binding pocket of the STAT3-SH2 domain, the compounds C3, C30, and C188 were found to be active in inhibiting IL-6-mediated phosphorylation of STAT3 with an IC_50_ of 91, 18 and 73 μM respectively [48]. As C188 was the most potent, Redell et al. performed similarity screening using C188 scaffold followed by 3-D pharmacophore analysis and identified more potent compounds. Among these second generation compounds, C188-9, inhibited G-CSF–induced STAT3 phosphorylation with low micromolar potency. IC_50_ of C188-9 in several AML cell lines ranged from 4.1 μM to 8.3 μM [49].

More recently Li and colleagues developed a novel STAT3-SH2 dimerization inhibitor by utilizing in-silico site-directed fragment-based drug design. They utilized naphthalene-5,8-dione-1-sulphoneamide fragment of compound LLL12 as binding moiety to pTyr705 of STAT3-SH2 domain and linked it to a dimethyl amine with an R group. Based on differences in R group they developed 5 different compounds. One of these compounds (LY5, IC_50_ 0.5-1.39 μM for U2OS and RD2 cell lines) found to be more potent than control (LLL12), easy to synthesize and possessed more drug-able properties [50].

Unfortunately, these STAT3 inhibitors are still not potent enough or drugable to be examined in clinical studies.

### Inhibitors targeting STAT5-SH2 binding domain

Similar to the development of STAT3 inhibitors, Berg et al. screened large small-molecule libraries in search of compounds that can modulate SH2 domain of STAT5. Out of 17,298 compounds, they found chromone-derived nicotinyl hydrazone (Figure 3e) as the most potent molecule disrupting the linkage between peptide, 5-carboxyfluorescein-GY(PO3H2)LVLDKW, derived from the erythropoietin (EPO) receptor, and the SH2 domain of STAT5b. It was 10 times more potent in inhibiting STAT5b-SH2 interaction (IC_50_ = 47 μM) than STAT3 (IC_50_ > 500 μM). This compound also inhibited IFNα stimulated STAT5 tyrosine phosphorylation in lymphoma (Daudi) cells. However, high concentration of the compound (100-200 μM) was required [10,51].

In an effort to develop more potent in vivo STAT5-SH2 inhibitor, Gunning et al. investigated the application of small molecule scaffolds targeting STAT5-SH2 domain. They performed in-silico screening of NCI library of small molecules, and showed that salicylic acid containing compounds effectively binds the STAT5-SH2 domains. Subsequently they accessed and screened their previously designed salicylic acid containing STAT3-SH2 domain binding library of compounds to find potent STAT5 inhibitors. From their rationally designed privileged structures, they identified two compounds (BP-1108 and BP-1075) to be most potent in vivo inhibitors of STAT5 in MV-4-11 and K562 leukemia cell lines. Their lead agent BP-1108 (IC_50_ = 17 μM for K562 cell line) also down- regulated STAT5 dependent genes, including *C-myc, cyclin D1, cyclin D2, MCL-1*[52].

### Inhibitors modulating STAT interaction with nuclear material

Platinum compounds are known to form DNA adduct and cause cytotoxic effects. Compounds like CPA-1, CPA-7 can disrupt STAT3 ability to bind DNA leading to apoptosis in STAT3 dependent human breast and colon cancer cell lines [53]. Another platinum compound, IS3 295 (Figure 3) inhibit STAT3 binding to its DNA response element (*in vitro* IC_50_ = 1.4 μM). It leads to cell cycle arrest, inhibition of proliferation with induction of apoptosis in human breast (MDA-MB-435, MDA-MB-468, and MDA-MD-231), pancreatic (Panc1), prostate (DU145), lung (non-small cell- A549) cancers and multiple myeloma (U266) cell lines [54]. As opposed to cisplatin, direct modification of DNA is not necessary for IS3 295 to inhibit STAT3-DNA interaction. Also, it binds with both inactive STAT3 monomers and active dimmers and blocks the binding of the later with DNA. These compounds possess minimal or no activity to inhibit STAT5-DNA interaction.

Another potent compound believed to inhibit STAT3 to DNA binding is Galiellalactone derived from ascomyecete, *Galiella rufa*. It induced apoptosis in hormone refractory prostate cancer cells in mouse xenograft when administered via daily intraperitoneal injection for 3 weeks. Galiellalactone inhibited STAT3-mediated luciferase activity (IC_50_ ≈ 5 μM) and reduced the relative mRNA expression of *Bcl-xL* and *Mcl-1*[55].

Covalent modification of histone proteins by acetylation and deacetylation of lysine residues modulate “opening” and “closing” of chromatin architecture for transcription. Acetylation of lysine residues neutralize the charges and recruit proteins containing bromodomains that are modules in DNA binding proteins. So far more than 60 bromodomains have been discovered in 46 different human proteins [56]. All bromodomain modules in these proteins share a highly conserved fold comprising left-handed bundle of four alpha helices, which make a deep hydrophobic cavity to house acetylated lysine residues on histone tails. BET bromodomain is the subfamily of bromodomain proteins comprising several important bromodomain like BRD2, BRD3, BRD4 and BRDT. JQ1 (Figure 3g), a small molecule that enters into the deep pockets of these bromodomains and hamper their interaction with chromatin and functioning of transcription complex thereof. Therefore it has the potential to inhibit the functioning of several transcription activators that utilize bromodomains as coactivator. By the same token, transcription of several oncogenes can also be decreased if their transcription initiation begins with bromodomain interaction e.g. *MYC*[57]. Liu S et al. studied lymphocytic leukemic cells with high STAT5 activity and treated them with JQ1. As BRD2 acts as co-activator of STAT5 dependent transcriptional activity, JQ1 was able to effectively inhibit STAT5-dependent transcription [58]. From this study one can predict that STAT5 driven cancers and diseases can be one of the several potential targets of JQ1 or related compounds.

### Oligonucleotides targeting STAT pathway

Advances in molecular biology not only have enabled the understanding of molecular basis of diseases but also provide tools to improve therapy. Some of these tools have been very promising in modulating STAT pathway and facilitating the development of new drugs for clinical application (Table 2).

**Table 2:** ***In vitro *** **and *in vivo *** **studies of nucleotide based STAT inhibitors**

**Nucleotide based Agent (Ref)**	**Cancer type studied *in vitro *** **(cell lines)**	**Cancer type studied *in vivo *** **(cell lines)**	**Clinical trial**
STAT3 ODN (59, 60, 61, 62, 63)	Head & Neck Squamous cell carcinoma (1483), Lung cancer (A549), Colon cancer (SW480), Glioma (U251, A172)	Lung (A549)	
Anti-Sense (AZD9150) (70, NCT01839604)			Non-Hodgkin lymphoma,Hepatocellular carcinoma
STAT3-siRNA (72, 73, 74, 75, 76, 77, 79)	Breast (MCF7, MDA-MB-231); CML (K562); Glioblastoma (A 172, U251-GM) ; Oral SCC (HSC3, HSC4, KB, GFP-SAS), Epidermoid carcinoma ( STAT3siRNA coupled with mAb to Lewis-Y antigen (A431); CTCL (Hut78)	Breast (MCF7, MDA-MB-231)	
STAT3-G-Quartet (82, 83, 84)	Prostate (PC-3, DU145, LNCap), Breast (MDA-MB-468)	Prostate (PC-3, DU145), Breast (MDA-MB-468)	
STAT5-ODN (64)	Chronic Myeloid Leukemia (K562)		
STAT5- siRNA (72, 81)	CML (K562), Hepatocellular (SMMC7721),	Hepatocellular (SMMC7721), Pancreatic cancer (HPAF-11)	

*Abbreviations*: *Ref*, references.

### Decoy oligonucleotides

Decoy Oligonucleotide (ODN) - a form of oligonucleotide based approach in which synthetically derived *cis*-element, usually double stranded 10–20 base pair sequences, is transfected into cells. These ODNs then bind with transcription factor’s DNA binding domain (e.g. STAT3-DNA binding domain) to prevent their interaction with DNA response element. This strategy effectively attenuates specific gene expression. Specific ODN has been studied for STAT3 and STAT5 inhibition in various cancer cell lines including K562, U251, A172, A549, and SW480 in vitro and in mice xenograft model of Lung cancer [59-64]. Therapeutic success of this approach relies on effective entry and stability of decoy oligonucleotide in the cells. Modification of phosphodiester bonds such as methyl phosphate, phosphoramidite, or methyl phosphonate and utilization of suitable gene transfer techniques successfully overcame many of these issues [65,66]. In addition, an experimental study supported the safety of STAT3 ODN in non-human primates [67]. ODN compounds have not progressed to clinical trials so far.

### Antisense oligonucleotide

Majority of antisense nucleotide drugs bind to messenger RNA (mRNAs) and inhibit the production of disease-causing proteins. Several antisense oligonucleotide (ASO) based drugs are in various phases of clinical trials. Mipomersen sodium (kynamro), a sequence of 20 nucleotides complementary to mRNA of apolipoprotein B-100, got FDA approval in January 2013 as adjunct to lipid lowering therapy [68,69].

AZD9150 (ISIS-STAT3Rx or ISIS 481464), a synthetic ASO against STAT3, underwent phase I evaluation in patients with advanced lymphoma and solid tumors. Investigators reported preliminary findings on 15 patients who were heavily treated in the past. Six patients with advanced lymphoma (3 DLBCL, 2 Hodgkin’s lymphoma, 1 mantle cell lymphoma) and 9 patients’ with solid tumors participated in this study. Thrombotic microangiopathy was the dose limiting toxicity in one patient and the dose of 2 mg/kg weekly after loading dose was recommended for phase II evaluation. 2/3 DLBCL patients demonstrated more than 50% reduction in tumor size. No responses were observed in any of the patients with solid tumors. Phase 2 study is currently ongoing [70] [NCT01563302].

Recently, a group from South Korea initiated another phase I trial utilizing AZD9150 in patients with advanced hepatocellular carcinoma [NCT01839604].

### RNA interference

RNA interference (RNAi) is a natural post-transcriptional gene-silencing mechanism to turn off unwanted genes. The process is initiated by the presence of double stranded RNA, not a constituent of a normal cell cytoplasm. The dsRNAs are cleaved by an endonuclease named dicer into 20–25 nucleotide dsRNA referred to as Short or Small Interfering RNAs (siRNAs). RNA-induced silencing complex separates the two strands, and one of these strands then serves as a guide for sequence-specific degradation of homologous mRNA. This mechanism was initially utilized to study gene function by silencing it. However, it can potentially be used clinically to knockout genes causing disease of interest.

Utility of this approach is limited because transfected RNAs have very short lives. This therefore requires frequent administration of siRNA in to the cells. Using DNA directed RNA interference technique, a short hairpin RNA (shRNA, a double stranded RNA) is expressed in the cell after insertion of a DNA construct in to the nucleus. These shRNAs then enter RNAi pathway. In this strategy gene silencing lasts for as long as the cell continues to produce its own shRNA [71].

This strategy is under evaluation in several clinical trials for the treatment of several diseases including cancers [NCT01591356, NCT00363714, NCT00689065, NCT00938574]. However, data regarding siRNA targeted silencing of STAT genes for cancer therapy are limited to in vitro studies and in vivo studies of animal models only [72-81].

### G-quartets

G-rich (Guanine rich) oligonucleotides can form inter- or intra-molecular four-stranded structures, called as G-quartets. G-quartets arise from the association of four G-bases into a cyclic Hoogsteen H-bonding arrangement. They have been shown to play vital role in several cellular processes. G-quartet–forming oligonucleotides can block STAT3 activity by targeting STAT3 proteins and inhibit their ability to bind DNA [82]. Jing and colleague demonstrated the efficacy of this strategy by utilizing T40214, a G-quartet, in vitro and in mouse model against prostate cancer cell lines [83,84]. However, this approach has not reached in to clinical trial.

## Conclusion and future directions

JAK/STAT pathways are major signaling channels for transmitting extracellular signals into the nuclei of cells. Monoclonal antibodies have been developed to target the signaling molecules along the related pathways [85-87]. In addition, small molecule inhibitors of JAK and Kit are widely used in cancer therapy [88-93]. Inhibitors targeting STAT molecules, particularly STAT3 and STAT5, are under intensive studies. Relatively less favorable/ drugable properties of peptidomimetics shifted the search towards the development of small molecules. However, candidate compounds at this time are not potent enough to advance to the level of clinical trial. Further work is warranted in this regard. Molecular techniques employing oligonucleotide approach seem promising.

## Competing interests

The authors have no relevant competing interests.

## Authors’ contributions

DL and MF designed the study. All authors have contributed to data preparation, drafting and revising the manuscripts. All authors have read and approved the final manuscript.
